# Flavin-based metabolic cycles are integral features of growth and division in single yeast cells

**DOI:** 10.1038/s41598-018-35936-w

**Published:** 2018-12-21

**Authors:** Bridget L. Baumgartner, Richard O’Laughlin, Meng Jin, Lev S. Tsimring, Nan Hao, Jeff Hasty

**Affiliations:** 1 Booz Allen Hamilton, 8283 Greensboro Drive, Hamilton Building, McLean, VA 22102 USA; 2 University of California, San Diego, Department of Bioengineering, La Jolla, CA 92093 USA; 3 BioCircuits Institute, University of California, San Diego, La Jolla, California, USA; 4 Molecular Biology Section, Division of Biological Science, University of California, San Diego, La Jolla, California USA

## Abstract

The yeast metabolic cycle (YMC) is a fascinating example of biological organization, in which cells constrain the function of specific genetic, protein and metabolic networks to precise temporal windows as they grow and divide. However, understanding the intracellular origins of the YMC remains a challenging goal, as measuring the oxygen oscillations traditionally associated with it requires the use of synchronized cultures growing in nutrient-limited chemostat environments. To address these limitations, we used custom-built microfluidic devices and time-lapse fluorescence microscopy to search for metabolic cycling in the form of endogenous flavin fluorescence in unsynchronized single yeast cells. We uncovered robust and pervasive metabolic cycles that were synchronized with the cell division cycle (CDC) and oscillated across four different nutrient conditions. We then studied the response of these metabolic cycles to chemical and genetic perturbations, showing that their phase synchronization with the CDC can be altered through treatment with rapamycin, and that metabolic cycles continue even in respiratory deficient strains. These results provide a foundation for future studies of the physiological importance of metabolic cycles in processes such as CDC control, metabolic regulation and cell aging.

## Introduction

Oscillations underlie a wide variety of biological phenomena. Their unique dynamical characteristics allow organisms across diverse kingdoms of life and at multiple length scales to perform a myriad of complex functions such as timekeeping^1^, resource allocation and sharing^2^, as well as coordinated behavior^3^. At the level of single -cells, the networks of interacting genes and proteins that generate oscillatory behavior have traditionally been the focus of investigation^1,4–7^. However, it is becoming increasingly clear that metabolic processes are also capable of periodic behavior, and that these oscillations may be integral parts of core biological processes such as glycolysis^8,9^, the cell division cycle^10–12^ and circadian rhythms^13,14^.

One of the most well-studied examples of metabolic oscillations is known as the yeast metabolic cycle (YMC). Since its initial observations about 50 years ago^15,16^, the YMC has come to be known as the bursts of respiratory metabolism and oxygen consumption by synchronized cultures of budding yeast growing in a nutrient-limited chemostat environment^17–19^. It has been shown that these oscillations correspond to a global coordination of cellular activity, where specific stages of the dissolved oxygen oscillations are associated with the expression of certain genes, the accumulation of distinct metabolites and progression through different phases of the cell division cycle^18,20,21^. Yet, despite the importance of these findings, the extent to which the many features of the YMC are recapitulated at the single-cell level remains to be determined. Answering these questions is made all the more difficult by the fact that different experimental set-ups can lead to markedly different observations about the period of the metabolic cycle and its relationship to the cell division cycle. For example, varying the strain background and chemostat conditions can lead to YMC periods ranging from 40 minutes^17,19^ to 5 hours^18^, and the YMC can even oscillate multiple times per cell cycle^22^ in specific deletion mutants or possibly disappear altogether at certain dilution rates^23^. Indeed, answering questions about the biological basis of metabolic cycles is challenging using synchronized cultures because it is difficult to decouple perturbations that affect cycling from those that merely prevent synchrony. As such, studies that could directly observe the dynamics of metabolism in single yeast cells would circumvent many of these challenges and greatly facilitate understanding of the mechanisms that generate the YMC.

Toward this end, seminal work by Papagiannakis *et al*.^12^ demonstrated the existence of metabolic cycles in the form of NAD(P)H and ATP oscillations in unsynchronized single yeast cells growing in a microfluidic device. A critical finding from their work was that cell cycle progression was synchronized with and gated by the metabolic cycle^12^ in multiple different media conditions. This distinguishes these oscillations from previously described glycolytic oscillations in single yeast cells, which are associated with NADH^8,9^ and pH^9^ oscillations that have periods of approximately one minute^8,9^ and appear to not manifest in all conditions^24,25^ or in all cells of a population^9,26^. While the work by Papagiannakis *et al*.^12^ establishes the existence of metabolic oscillations in single cells and supports previous findings^27–29^ along this line, questions remain about the degree to which other features of the YMC, as observed in synchronized chemostat cultures, are also extant at the single-cell level.

Here we used novel microfluidic technology and time-lapse fluorescence microscopy to directly observe and quantitatively characterize the dynamics of metabolic cycling in unsynchronized single yeast cells. To accomplish this we monitored changes in cellular redox state via endogenous flavin fluorescence. We observed clear and robust oscillations that displayed the expected phase relationships relative to the cell division cycle as reported from YMC chemostat studies^18,20,23^. Our results indicate that the metabolic cycle is a robust oscillator that typically couples one-to-one to the cell division cycle across four different nutrient conditions, however, we found that treatment with rapamycin can alter phase synchrony between these two oscillators, leading to multiple metabolic cycles per CDC and increased variability of metabolic cycling. Additionally, in contrast to the chemostat studies of the YMC, we found that cellular respiration is dispensable for metabolic cycling, as flavin oscillations persisted in deletion mutants impaired in oxidative phosphorylation. These results are in general agreement with the previous findings from Papagiannakis *et al*.^12^, and demonstrate that another critical cellular metabolite, flavin, also oscillates during the cell division cycle. Our work provides new insights into metabolic cycling in budding yeast and provides a foundation and methodology for future studies of its role at the level of single cells.

## Results

### Tracking metabolic cycles in unsynchronized single cells via endogenous flavin fluorescence

To investigate metabolic cycling in unsynchronized single cells, we designed a custom microfluidic device to minimize the accumulation of possible synchronizing agents such as hydrogen sulfide and acetaldehyde. At the high cell densities experienced in chemostats, such compounds are thought to accumulate in the media and synchronize nearby cells by phase shifting the YMC^19,30^. Our microfluidic platform minimizes such effects by trapping single cells in physically isolated monolayer columns (Fig. S1), thus preventing cell-to-cell communication. Experiments were conducted with the CEN.PK strain previously shown to produce metabolic cycles with a period of several hours in chemostat environments^18^.

As a readout of metabolic cycling, we used the natural redox-sensitive fluorescence of riboflavin, flavin mononucleotide (FMN) and flavin adenine dinucleotide (FAD). It has been shown that the abundance of these molecules is highly periodic during the YMC in chemostats, exhibiting a near anti-phase relationship with the dissolved oxygen concentration^19,20^. We reasoned that we could utilize flavin molecules as dynamic reporters of metabolic cycling in single cells because of their unique fluorescent properties; they fluoresce in the visible spectrum (peak excitation/emission: 450/535 nm)^31^ and their reduced equivalents (FADH_2_ and FMNH_2_) have negligible fluorescence in this range^32^, allowing their oxidized and reduced states to be distinguished. This approach obviates the need for expression of fluorescent proteins as YMC reporters, which may not be dynamic enough to observe multiple cycles or could influence the natural period.

**Figure 1:**
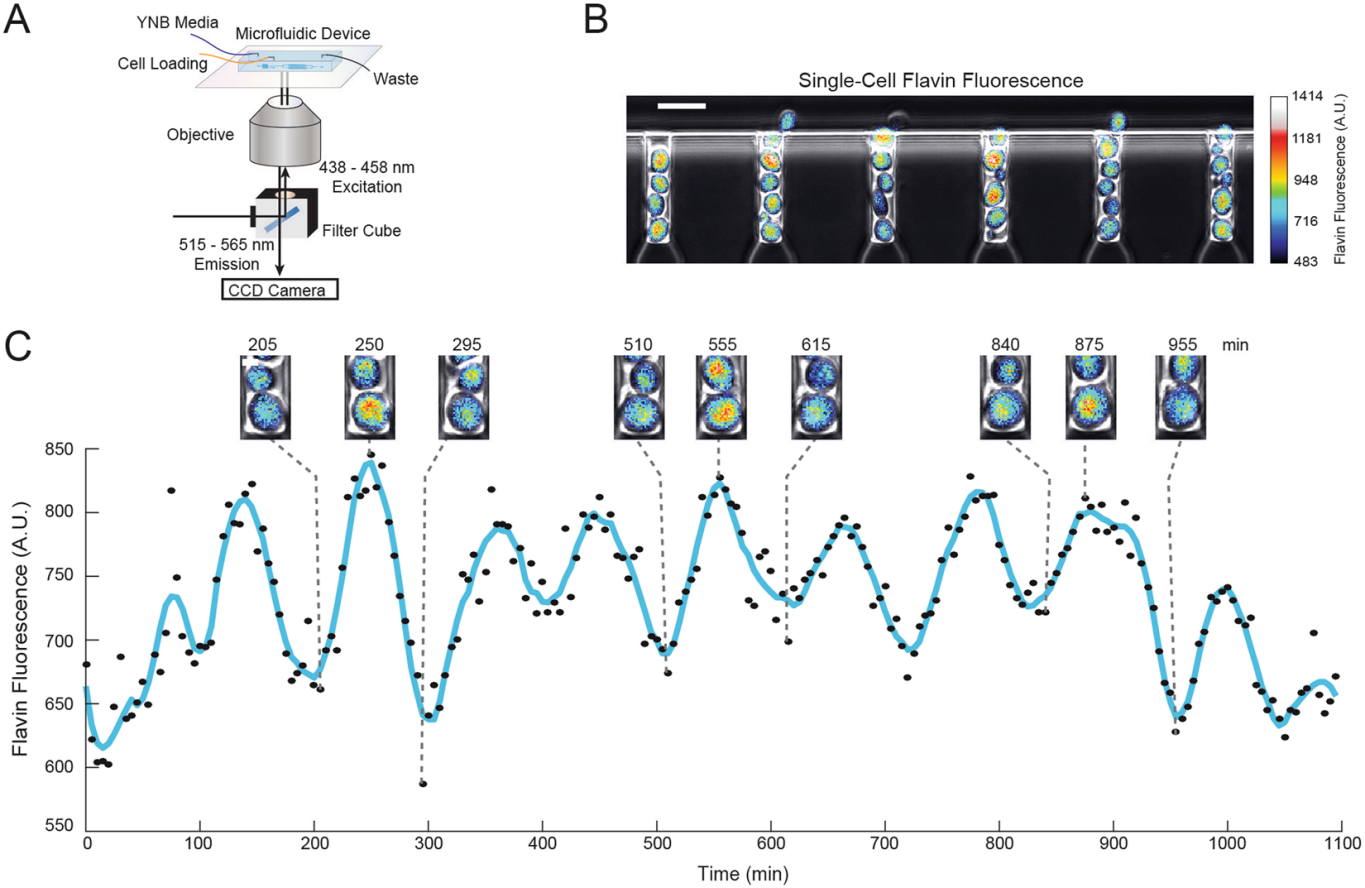
Flavin oscillations in single yeast cells. (**A**) Experimental setup for observing flavin oscillations in single cells. (**B**) Snapshot of flavin fluorescence in single cells growing in the microfluidic device during an experiment. The phase and flavin fluorescence channels were overlayed and false coloring using the ImageJ^50^ ‘royal’ colormap was applied to the flavin channel in order to increase visual contrast for presentation. The colorbar to the right indicates the intensity of the measured flavin signal^50^. The scale bar is 10 *μ*m. (**C**) Single-cell trajectory of measured flavin fluorescence including snapshots of the cell undergoing oscillations at the labeled time points. The color bar is the same as in panel B. The scale bar is 2 *μ*m.

We used a customized filter cube (excitation at 438–458 nm and emission at 515–565 nm) to image flavin fluorescence during time-lapse microscopy experiments (Fig. 1A). The flavin fluorescence signal was clearly visible in individual cells growing in the microfluidic device (Fig. 1B) and exhibited good dynamic range, increasing more than 2-fold with the addition of hydrogen peroxide to the media (Fig. S2). By imaging and tracking individual cells growing in minimal yeast nitrogen base (YNB) media, we observed strikingly clear oscillations in flavin fluorescence during multiple cell divisions (Fig. 1C, Movie S1).

**Figure 2:**
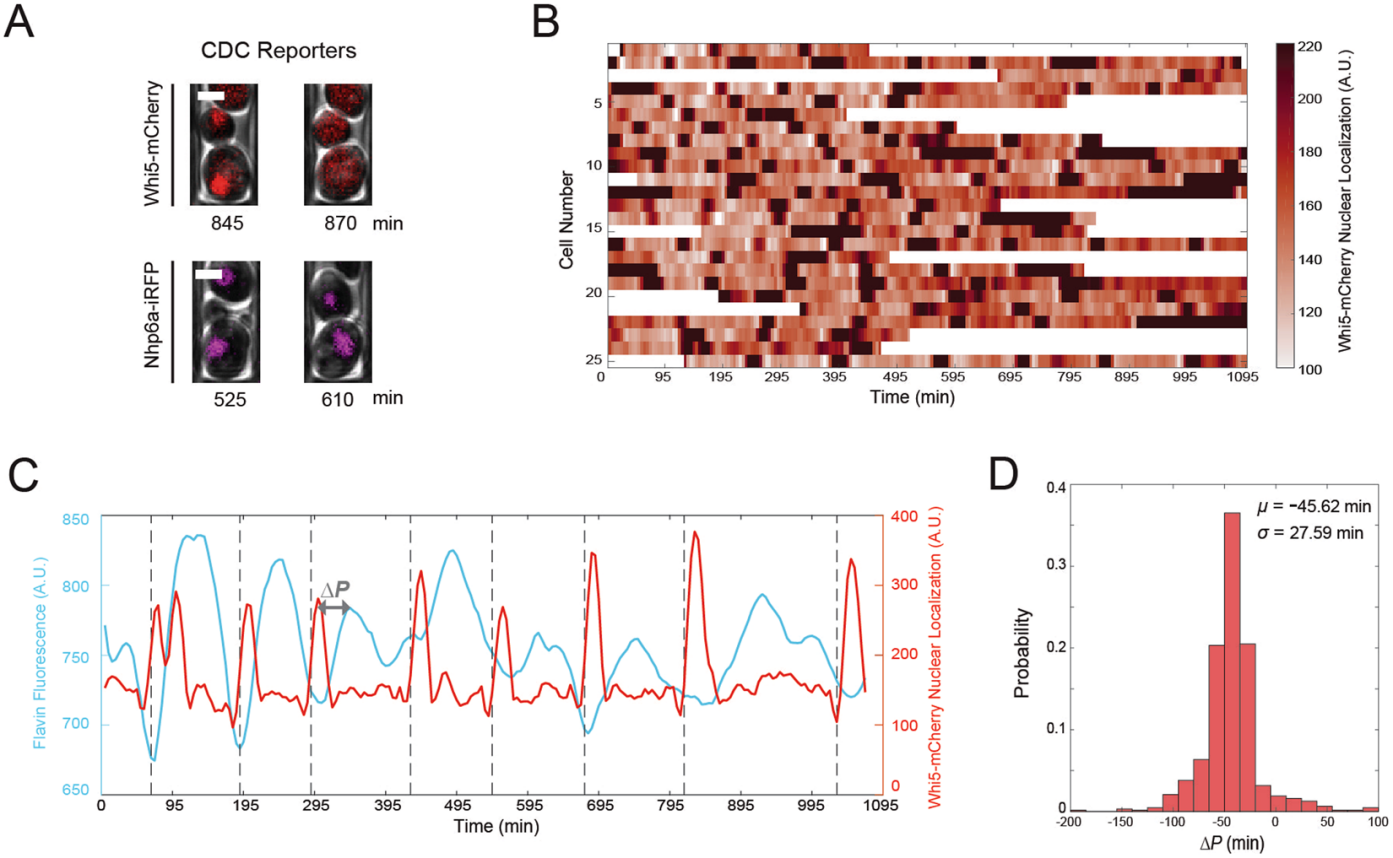
Tracking the dynamics of flavin fluorescence relative to the cell division cycle. (**A**) Snapshots of the dynamics of fluorescent reporters of the cell division cycle in single cells. Fluorescently tagged proteins Whi5-mCherry and Nhp6a-iRFP were used for demarcating the early and late phases of the cell cycle respectively. All scale bars are 2 *μ*m. (**B**) Representative heatmap of Whi5-mCherry nuclear localization from 25 single cells, demonstrating a lack of CDC synchrony. (**C**) Example trace of flavin fluorescence and the Whi5-mCherry nuclear localization signal in the same single-cell. Pulses of the Whi5-mCherry signal correspond to nuclear localization. The lag time between the Whi5-mCherry and flavin peaks was denoted as Δ*P* and was calculated as the difference between the time of the Whi5-mCherry peak and the flavin fluorescence peak within each cell division cycle. The black dotted vertical lines indicate separation of the mother and daughter nuclei as visualized by the Nhp6a-iRFP reporter. (**D**) Distribution of the time difference between flavin and Whi5-mCherry peaks (*n* = 156 cells, the mean (*μ*) and standard deviation (*σ*) for the distribution are Δ*P* = −45.62 ± 27.59 minutes).

Having observed oscillatory flavin dynamics in single cells, we next sought to determine the extent to which these oscillations were related to the metabolic cycles reported in chemostat cultures. The cell division cycle has been shown to be highly correlated with phase specific processes during the YMC in synchronized cultures, with budding occurring near the trough of the oxygen oscillations in the chemostat^18,23^. Further, Papagiannakis *et al*.^12^ demonstrated a strong coupling between NAD(P)H and ATP oscillations and the cell division cycle in single cells. Here, we decided to explore the quantitative relationship between the observed flavin oscillations and the CDC. To accomplish this we constructed strains with fluorescent reporters that allowed us to monitor different phases of the cell division cycle. We analyzed the relationship of flavin oscillations to the early part of the cell cycle by tracking the nuclear localization of Whi5-mCherry (Fig. 2A top row), which exits the nucleus before budding^33^. We also tracked how flavin oscillations related to the late part of the cell cycle by monitoring the separation of the mother and daughter nuclei using NHP6a-iRFP as a nuclear marker (Fig. 2A bottom row). By tracking Whi5-mCherry nuclear localization, we verified that cells growing in the microfluidic device were not synchronized (Figs 2B, S3), and subsequently moved to analyzing the relationship between the flavin oscillations and the early part of the cell division cycle. We found that the flavin signal reached its peak shortly after Whi5-mCherry exited the nucleus (Figs 2C, S4A). Peak nuclear localization of Whi5-mCherry occurred approximately 45 minutes before the peak of the flavin signal (Fig. 2D). The troughs of the flavin oscillations closely corresponded with the separation of the mother and daughter nuclei (Fig. 2C dashed lines). The timing of the flavin peak relative to the CDC is consistent with findings from synchronized chemostat cultures^20^ and also closely matches the timing of peak NAD(P)H fluorescence observed by Papagiannakis *et al*.^12^

These findings validate our use of flavin fluorescence as a reliable reporter of metabolic cycling in single cells. Further, by using fluorescent reporters to monitor the cell division cycle we were able to demonstrate that cells growing in the microfluidic device are not synchronized and that the flavin dynamics, with respect to the CDC, in single cells are similar to those observed for synchronized chemostat cultures^20,23^. Having validated our methodology and uncovered evidence of metabolic cycling, we next sought to quantify properties of the metabolic cycle and further explore its relationship with the cell division cycle in single cells.

### Metabolic cycles are synchronized with the cell division cycle across different nutrient conditions

We then turned to investigate the dynamics of the metabolic cycle and its coupling to the cell division cycle under different nutrient environments. First, we measured flavin oscillations in unsynchronized cells growing in media with a range of yeast nitrogen base (YNB) concentrations. For media preparation, YNB containing ammonium sulfate as a nitrogen source, as well as other nutrients such as vitamins and minerals, was diluted relative to 1X YNB media. We also tested the effect of varying the nitrogen source alone by analyzing cells growing in 1X YNB media with urea as the nitrogen source, a non-preferred option for yeast^34^. In all four media conditions the level of glucose was kept constant at 1%.

**Figure 3:**
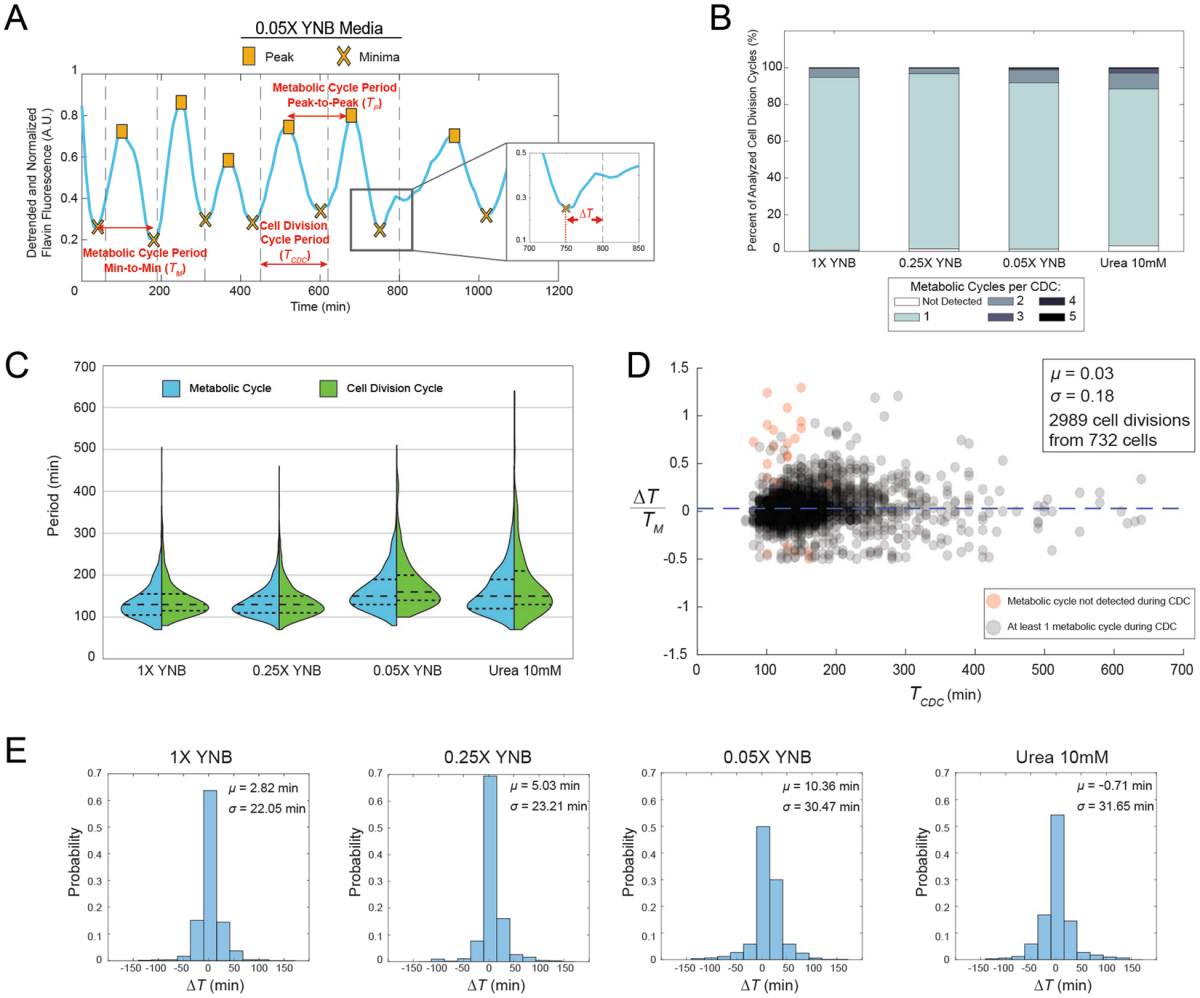
Phase synchronization and coupling between the metabolic cycle and CDC in different nutrient environments. (**A**) Summary of the information collected from each single-cell. Across four media conditions we recorded the peaks and troughs (yellow squares and ‘X’ marks respectively) of normalized and detrended metabolic cycles, the separation of the mother and daughter nuclei (black dotted lines), and the time difference between each mother-daughter nuclear separation event and the nearest metabolic cycle trough. Thus for each condition we could quantify the metabolic cycle period (both the peak-to-peak (*T*_*P*_) and min-to-min (*T*_*M*_) period), the CDC period (*T*_*CDC*_) and the coupling or lag (Δ*T*) between the metabolic cycle and CDC. (**B**) Number of observed metabolic cycles occurring during each cell division. (**C**) Split violin plots of the distributions of peak-to-peak periods (*T*_*P*_) of the metabolic cycle and the CDC periods (*T*_*CDC*_) for cells in each nutrient condition. Dotted lines represent the quartiles of the distributions. (**D**) Single-cell data of each Δ*T* scaled by the metabolic cycle period (min-to-min period *T*_*M*_) versus the CDC period (*T*_*CDC*_) shows that regardless of the length of the cell division cycle, division is completed near a metabolic cycle trough in most cases. Data from all four media conditions is displayed. The Δ*T* was calculated for every CDC in each cell, a total of 2989 cell divisions from 732 individual cells. The mean Δ*T*/*T*_*M*_ value is *μ* (blue dashed line) and *σ* is the standard deviation. (**E**) Distributions of the absolute lag time Δ*T* for each media condition. The number of cells analyzed for the 1X YNB, 0.25X YNB, 0.05X YNB and 10 mM urea conditions are as follows: 156 cells, 225 cells, 175 cells and 176 cells.

Metabolic cycles robustly persisted in all nutrient environments (multiple sample trajectories can be found in Figs S4 and S5), with more than 150 cells being analyzed in each experiment. For cells in each media condition, we determined the period of the metabolic cycle and the cell division cycle (Fig. 3A, see Fig. S6 for estimation of metabolic cycle periods using autocorrelation analysis). The relative coupling between the metabolic cycle and CDC was quantified by measuring the lag time between the trough of the metabolic cycle and the separation of the mother and daughter nuclei at the end of each CDC. This quantity was termed Δ*T* (Fig. 3A).

Across all media conditions one metabolic cycle accompanied each CDC in at least 85% of all cases (Fig. 3B). As nutrient quality of the media decreased, the mean period of the metabolic cycle increased. The 1X YNB and 0.25X YNB conditions gave similar mean metabolic cycle periods of ~136 minutes and the less nutrient-rich 0.05X YNB and 10 mM urea conditions both gave mean periods of ~163 minutes (Table S3). The period of the metabolic cycle and the cell division cycle were similar for all nutrient conditions as well (Fig. 3C and Table S3). For the 1X YNB and 0.25X YNB conditions the median CDC and metabolic cycle times were both 130 minutes (Fig. 3C). For the 0.05X YNB condition the median CDC time was 160 minutes, while the median metabolic cycle time was 150 minutes, and both the median CDC and metabolic cycle times were 150 minutes in the 10 mM urea media (Fig. 3C). We note that the median and first and third quartiles of the CDC period distributions are slightly higher in some cases than those for the metabolic cycle (Fig. 3C). This is largely due to the fact that the metabolic cycle can occasionally oscillate multiple times before division (Fig. 3B), particularly when CDC progression is stalled. Taken together, these results suggest that the metabolic cycle can function over a range of periods, and that it is tuned along with the CDC in accordance with the nutrient conditions of the environment.

Synchronization of the metabolic cycle and cell division cycle was further established by analyzing the Δ*T* values for cells in each nutrient condition. Regardless of the length of the CDC, separation of the mother and daughter nuclei occurred, in almost all cases, near a metabolic cycle trough in all four media conditions (Fig. 3D,E). These findings suggest that the CDC is strongly coupled to and gated by the metabolic cycle; division of the mother and daughter nuclei occurs at a relatively fixed time near a trough of the metabolic cycle. This is true even for prolonged cell division cycles where the metabolic cycle can oscillate multiple times before division occurs. Further, these results demonstrate that carbon source limitation, as commonly used in the literature^18,23^, is not a requirement for generating or modulating the metabolic cycle.

### Rapamycin perturbs the phase synchronization of the metabolic cycle and CDC

The strong connections we found between the metabolic cycle and CDC in different media conditions led us to investigate ways in which we could possibly alter the relationship between these two oscillators. Since previous work in synchronized cultures showed that the addition of rapamycin can phase shift metabolic cycles into a prolonged reductive phase^21^, and that rapamycin is a known CDC inhibitor^35^, we reasoned that it could be used to perturb the connection between the metabolic cycle and the cell division cycle.

**Figure 4:**
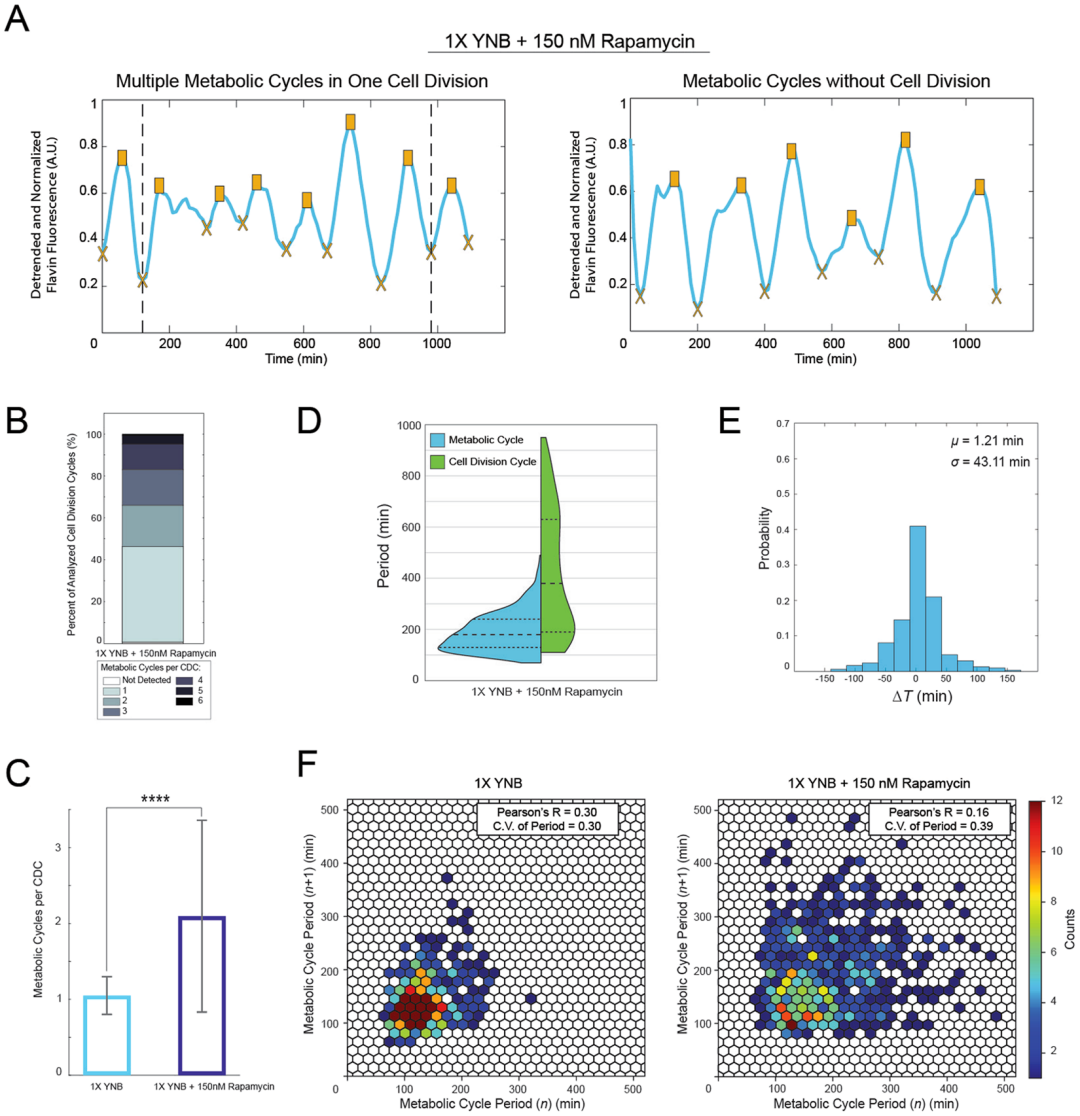
Rapamycin alters the periodicity of the metabolic cycle and its phase synchronization with the CDC. (**A**) Examples of metabolic cycles in cells treated with 150 nM rapamycin. There was an increased occurrence of multiple metabolic cycles during each cell division cycle (left panel). Further, some cells displayed metabolic cycles in the absence of complete CDC progression (right panel). (**B**) Number of observed metabolic cycles occurring during each cell division cycle. Metabolic cycles not occurring during a division event were not included here. (**C**) Comparison of the number of metabolic cycles during each CDC in 1X YNB media and 1X YNB media with 150 nM rapamycin, showing a significant increase for the rapamycin treated cells (Kolmogorov-Smirnov test; ****P < 0.0001, *P* = 2.89 × 10^−25^). Error bars are the standard deviation. (**D**) Split violin plot of the metabolic cycle (*T*_*P*_) and CDC (*T*_*CDC*_) periods for rapamycin treated cells. The number of cells used to calculate metabolic cycle periods was 180 and the number used to calculate CDC periods (cells that divided at least twice) was 85. Dotted lines represent the quartiles of the distributions. (**E**) Distribution of Δ*T* values for rapamycin treated cells that divided at least once during the experiment (*n* = 151 cells). Δ*T* is defined the same as in Fig. 3. (**F**) Hexagonal binning plot of each peak-to-peak metabolic cycle period *n* versus the next period *n* + 1. Colors correspond to the number of values within each hexagon.

When cells were grown in 1X YNB media in the presence of 150 nM rapamycin around half of the cells continued dividing but at a greatly reduced rate. In such cases we often observed multiple metabolic cycles occurring within a single cell division cycle (Fig. 4A left panel, see also Fig. S7 for additional trajectories and Movie S2). Other cells divided only once or were delayed in the early phases of the CDC and did not complete a full division during the course of our experiment, yet metabolic cycles were able to continue in these cells as well (Fig. 4A right panel, see Fig. S7 for additional trajectories and Movie S3). Overall, there was an increase in the number of metabolic cycles occurring in each cell division cycle. In 1X YNB without rapamycin about 90% of all cell divisions contained only one metabolic cycle (Fig. 3B) while this dropped to less than 50% with the addition of rapamycin (Fig. 4B). Cells exposed to rapamycin had a significantly greater (*P* < 0.0001) average number of metabolic cycles during each CDC than cells in the 1X YNB condition without rapamycin (Fig. 4C).

Compared to the cells in 1X YNB media without rapamycin (Fig. 3C), the periods of the metabolic cycle and cell division cycle were much longer in the rapamycin exposed cells (Fig. 4D). Indeed the mean metabolic cycle period of the rapamycin treated cells was more similar to that of cells grown in 0.05X YNB media (191.73 ± 75.41 min for rapamycin treated cells and 162.80 ± 47.52 min for cells in 0.05X YNB media). Although exposure to rapamycin increased the period of both the metabolic cycle and CDC, the metabolic cycle was not prolonged to the same extent as the cell division cycle. The mean CDC time in rapamycin was ~190% greater than without, while the mean metabolic cycle period was increased ~40% (Fig. 4D and Table S3). Interestingly, despite the differences in mean metabolic and cell division cycle periods, separation between the mother and daughter nuclei still occurred close to a metabolic cycle trough (Fig. 4E). Additionally, there was a loss of periodicity in the metabolic cycle, where successive period times became more irregular (Fig. 4F).

These findings demonstrate that rapamycin can affect the phase synchrony of the metabolic cycle and CDC. Under these conditions we observed more instances of multiple metabolic cycles occurring before a cell division event, however mother-daughter nuclear division at the end of the CDC remained near the metabolic cycle trough, just as in 1X YNB media without rapamycin. Furthermore, metabolic cycles could persist even without cells completing the cell division cycle. These results support the model proposed by Papagiannakis *et al*.^12^ where CDC progression is gated by an autonomous metabolic oscillator.

### Metabolic cycling continues in respiratory deficient mutants

Having established that metabolic cycles occur under multiple conditions and can be decoupled from the cell division cycle, we turned to studying the role of cellular respiration in metabolic cycling. Chemostat studies of the YMC have mainly used the dissolved oxygen content in the media as a readout of the YMC^17–19^. Indeed, cellular respiration has been viewed as instrumental to the function of these cycles, as the yeast metabolic cycle has sometimes been referred to as yeast respiratory oscillations^36–38^. However, given that such studies have been conducted using synchronized chemostat cultures, it remains unclear if oxidative respiratory metabolism is required for metabolic cycles to occur, or if that mode of metabolism is simply the most favorable for synchronizing the culture and generating oscillations.

**Figure 5:**
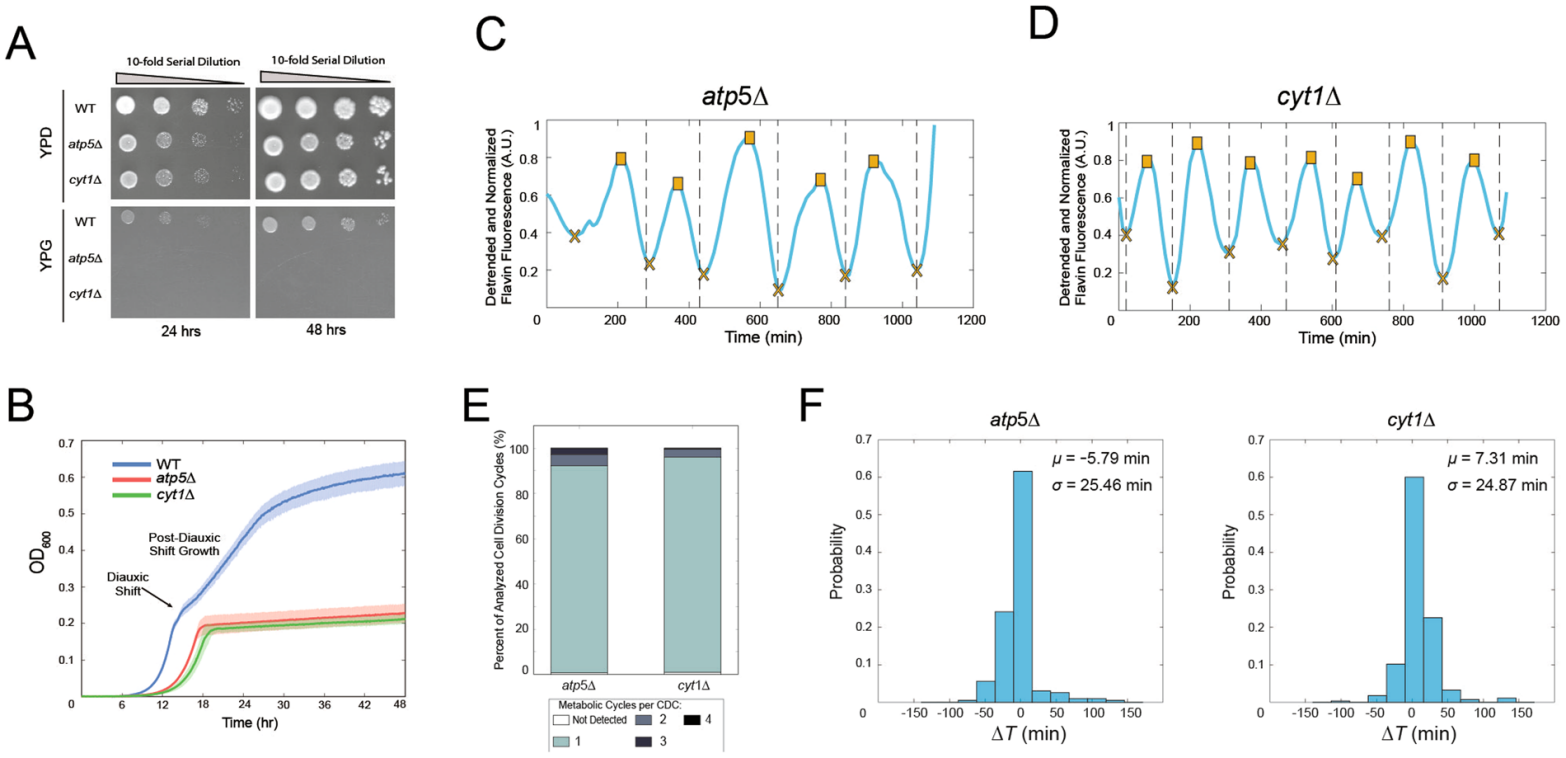
Metabolic cycles continue in respiratory deficient mutants. (**A**) The *atp5*Δ and *cyt1*Δ mutants do not exhibit noticeable growth on YPG plates containing the non-fermentable carbon source glycerol (bottom panels). Cells growing on YPD served as a control (top panels). (**B**) Growth curves demonstrating that cultures of *atp5*Δ and *cyt1*Δ strains display no post-diauxic shift growth, indicating an inability to conduct respiratory metabolism. Solid lines and shaded regions represent the means and standard deviations, respectively, from four replicates for each strain. (**C**) Representative metabolic cycling in the *atp5*Δ strain. (**D**) Representative metabolic cycling in the *cyt1*Δ strain. (**E**) The number of metabolic cycles occurring per cell division cycle for each strain (*n* = 54 cells for *atp5*Δ, and *n* = 52 cells for *cyt1*Δ). (**F**) Distributions of Δ*T*, the time between the metabolic cycle trough and separation of the mother and daughter nuclei for each strain.

To address this question we tracked the dynamics of flavin fluorescence in the respiratory-deficient *atp5*Δ and *cyt1*Δ strains growing in 1X YNB media. The *ATP5* gene codes for a subunit of the ATP synthase complex^39^ while the *CYT1* gene codes for cytochrome *c*1^40^. Both are critical components of mitochondrial respiration, and both genes have been shown to be required for respiratory growth^41,42^. We verified that the *atp5*Δ and *cyt1*Δ strains in the CEN.PK background were respiratory deficient by growing cells on the non-fermentable carbon source glycerol. No growth of either strain was observed on YPG (yeast extract, peptone and 3% glycerol) plates over the course of two days (Fig. 5A). Further, *atp5*Δ and *cyt1*Δ cultures did not exhibit post-diauxic shift growth, also indicating an inability to carry out respiratory metabolism^42^ (Fig. 5B). Despite this, we continued to observe metabolic cycles in these strains (Fig. 5C,D and Movie S4 of the *cyt1*Δ strain). As in other media conditions tested (Fig. 3), predominantly one metabolic cycle occurred per CDC (Fig. 5E), and separation of mother and daughter nuclei was near a metabolic cycle trough (Fig. 5F). These results suggest that metabolic cycles are respiration independent, as neither their maintenance nor their coupling to the cell division cycle were significantly effected in the *atp5*Δ and *cyt1*Δ strains.

## Discussion

Elucidating the workings of biological processes often benefits from multiple methods of investigation. Through the use of microfluidics and time-lapse fluorescence microscopy we directly observed metabolic cycling in single yeast cells. These metabolic cycles operated across four different nutrient conditions, could exist synchronized and unsynchronized with the cell division cycle, continued without the need of significant cellular respiration and seemed to gate progression of the CDC. These findings both validate and expand the current understanding of metabolic cycles and build off of the work reported in foundational studies conducted with synchronized cultures^17,18,22,23,30,36,37^ and in single cells^12^. The unique insights provided in this work by analyzing single cells suggests that metabolic cycling is an integral feature of yeast metabolism that is not confined to any specific environmental condition.

With regard to the generality of metabolic cycles in different yeast strains, it is of particular interest that Papagiannakis *et al*.^12^ conducted their work in the YSBN strain background (derived from S288C^43^), while we carried out our work in the CEN.PK strain background. It has been shown that these two popular lab strains have key differences in their metabolism, particularly with respect to amino acid and protein synthesis as well as glucose catabolism, that manifest as differences in maximal specific growth rate^43^. Yet, despite these differences, metabolic cycles with similar periods were observed in both strains^12^. Together our two studies support the notion that metabolic cycling is a general phenomenon that occurs across different yeast strains and forms a system of coupled oscillators with the cyclin-dependent kinase machinery to drive cell division, as originally proposed by Papagiannakis *et al*.^12^.

The single-cell approach to studying metabolic cycles could offer a whole host of interesting research directions, as the dynamics of cellular metabolic activity can be studied in relationship to numerous other processes. Perturbations to chromatin regulation, cellular redox state and specific metabolic pathways have yielded interesting insights into YMC dynamics in synchronized cultures^44^ and such results could be greatly complemented by similar studies at the single-cell level. Hypotheses regarding the biological functions of the metabolic cycle, such as its possible role as a biological clock in the cell aging process^37^, could be investigated using the methods described here combined with microfluidic technology capable of studying dynamic processes during cell aging as recently described^45–47^. Additionally, it has been proposed that signaling networks such as target of rapamycin complex I (TORC1) and protein kinase A (PKA) could play important roles in metabolic cycling^11^. This hypothesis is supported by our data showing that rapamycin can slow the metabolic cycle (Fig. 4) and that respiratory related mitochondrial activity does not appear to play a critical role in generating or maintaining metabolic cycles (Fig. 5). As the TOR and PKA regulated processes of ribosome biogenesis and translation are both energetically costly and critically important for cell growth and entry into division^11,48^, a connection with the metabolic cycle is an intriguing possibility. Indeed, as metabolic cycles are studied in greater detail, they may come to be critically important for our understanding of how cells function, as they represent one more biological knob that can be dynamically modulated to optimize cellular fitness.

## Methods

### Yeast strains and growth conditions

In all experiments prototrophic strains derived from the haploid CEN.PK2-1c strain (MATa, trp1-289, his3Δ1, ura3-52, leu2-3_112) were used (Table S1). The CEN.PK2-1c strain was purchased through EUROSCARF (Accession Number: 30000A) and standard yeast integration vectors were used to repair auxotrophies (Table S1). The standard lithium-acetate method was used for transformations. Before experiments, cells were cultured overnight in 1X yeast nitrogen base (YNB) media that contained 6.7 g/L of Difco yeast nitrogen base without amino acids (Becton, Dickson and Company) and 1%(wt/vol) glucose (Sigma-Aldrich). The day of the microfluidics experiment the overnight culture was diluted in 1X YNB media to an OD600 of 0.05 and grown to a final OD600 between 0.2 and 0.6 before being loaded into the microfluidic device. In experiments where 0.25X YNB or 0.05XYNB media was used, yeast nitrogen base without amino acids was diluted to 0.25X or 0.05X as indicated in sterile filtered deionized water. Glucose was kept constant at 1%(wt/vol) in all media conditions. For the 1X YNB with 10 mM urea media, 1.7 g/L of Difco yeast nitrogen base without amino acids and without ammonium sulfate (Becton, Dickson and Company) was used and 1%(wt/vol) glucose (Sigma-Aldrich) was added. Urea (Sigma-Aldrich) was added to a final concentration of 10 mM and the pH was then adjusted to 5.4 to match that of the standard 1X YNB with ammonium sulfate. The rapamycin (Sigma-Aldrich) was added to a final concentration of 150 nM.

For assessing the respiratory growth of the WT, *atp5*Δ and *cyt1*Δ strains, overnight cultures were diluted to an OD600 of 0.3, 0.03, 0.003 and 0.0003, then spotted on YPD (1% bacto-yeast extract (Becton, Dickson and Company), 2% bacto-peptone (Becton, Dickson and Company), 2% glucose (Sigma-Aldrich)) and YPG (1% bacto-yeast extract (Becton, Dickson and Company), 2% bacto-peptone (Becton, Dickson and Company), 3% glycerol (v/v) (Sigma-Aldrich)) plates. Plates were incubated at 30 °C and imaged after 24 and 48 hours using a BioDoc-It Imaging System (UVP). To test for post-diauxic shift growth, overnight cultures were grown in YPD media, diluted to an OD600 of 0.2 and 0.5 *μ*l was added to wells of a 96 well flat bottom tissue culture plate (Falcon) containing 50 *μ*l YPD. There were four biological replicate wells for each strain. A blank YPD well containing no cells was included, and surrounding wells were filled with water to prevent evaporation during the experiment. The plate was placed in a Infinite 200 PRO plate reader (Tecan) and grown at 30 °C for 48 hours with 600 nm absorbance values being recorded every 5 minutes. For data analysis, the value of the blank well at each time point was subtracted from each well. The mean and standard deviation across the four replicates was plotted in Fig. 5B.

### Microfluidics and time-lapse microscopy

The microfluidic device used in this study was designed to trap short single rows of cells in individual rectangular traps that would minimized intercellular communication and provide good resolution of single cells. To accomplish this cell traps were 25 *μ*m by 5.25 *μ*m and approximately 4.7 *μ*m tall. Cell traps are open on both ends allowing media to flow through the traps and provide continuous nutrients to the cells. The device design (Fig. S1) was drawn in AutoCAD (Autodesk) and then printed on chrome glass masks (HTA Photomask). Standard procedures similar to those detailed by Ferry *et al*.^49^ for photolithography with SU8 (MicroChem) were used to pattern silicon wafers (University Wafer) with an EVG620 mask aligner (EV Group) and then make PDMS (polydimethylsiloxane, Dow SYLGARD) molds of the devices.

Log-phase cells as described above were loaded into the microfluidic device with the appropriate media. Images were taken on a Nikon Eclipse Ti inverted microscope with a CoolSnap HQ2 camera (Photometrics) and Lumencor SOLA system (Lumencor) fluorescent light source at 60X magnification using an oil immersion objective. The microfluidic device was surrounded by a plexiglass case that maintained the temperature at 30 °C throughout the experiment. Flavin fluorescence was measured using a customized filter cube (Semrock) that allowed excitation at 438–458 nm and emission at 515–565 nm. Exposure settings were 150 ms at 15% lamp intensity for flavin, 200 ms at 20% lamp intensity for mCherry and 100 ms at 15% lamp intensity for iRFP. For all fluorescent signals 2-by-2 binning was used. Images were acquired every 5 minutes for the 1X YNB experiment and every 10 minutes in all other experiments. The Whi5-mCherry background strain (yYMC1 from Table S1) was used for experiments in Figs 3 and 4. The Whi5-mCherry background strain (yYMC1 from Table S1) was used to create the *atp*5Δ and *cyt*1Δ strains (yYMC4 and yYMC5 from Table S1). For Fig. 5 the ‘WT’ strain in panels A and B is the Whi5-mCherry strain yYMC1. Experiments lasted approximately 18 hours for analyzing metabolic cycling in different nutrient environments (Figs 3, 4 and 5).

### Cell tracking and data analysis

Time-lapse image stacks were pre-processed in Image-J^50^ for background subtraction and registration. Fluorescence values of single cells were obtained with custom MATLAB (Mathworks) scripts for cell tracking. We tracked and analyzed one viable cell at the bottom of each trap. Most cells eventually bud in the direction opposite the trap opening and move up the trap, eventually exiting it. In these cases we often tracked cells for some time before they exited the trap until the automated tracking code or manual tracking was no longer accurate or convenient for that cell. Cells tracked for less than 4.5 hours were excluded from analysis. In all experiments cell masks generated by tracking code were manually inspected to ensure their accuracy, and manually edited when necessary. For period analysis, fluorescence trajectories were first detrended using a polynomial function of order 7 fit to the data. Each trace was then normalized by their maximum value and filtered using the MATLAB Savitzky-Golay filter. These smoothed trajectories were used for analysis where custom MATLAB scripts detected metabolic cycle peaks based on fixed and standardized criteria (Table S2). The period values were calculated by the code and obtained by determining the time between successive peaks (*T*_*P*_) or successive minima (*T*_*M*_). If a tracked cell appeared to become sickly or near death, we made special note of these cells and excluded from analysis cell division times that occurred one division before this and only considered fluorescence values that were five time points after this for peak and minima detection. For determining the cell division times, in all cases we manually recorded the separation times of the mother and daughter nuclei for each single-cell, with the division time being the time difference between each nuclear separation and the one that follows for the next division. For tracking the dynamics of Whi5-mCherry we followed the method used by Cai *et al*.^51^ for determining nuclear localization, where, for each cell mask, we took the difference between the averages of the top five brightest pixels and of all other pixels in the cell mask. This process gave fluorescence traces with spikes that corresponded well to Whi5-mCherry nuclear localization. The peak of the Whi5-mCherry nuclear localization signal was taken to be the maximum peak value within each cell cycle. The color map used in Fig. 2B and Fig. S3B is from Thyung *et al*.^52^ For quantifying the flavin fluorescence we took the mean intensity over the entire cell mask.

## Electronic supplementary material

Supplementary Information

Movie S1

Movie S2

Movie S3

Movie S4

## Data Availability

Data from this manuscript is available upon request from the authors.
