# Supplementary material for: Flavin-based metabolic cycles are integral features of growth and division in single yeast cells
Source: Sci Rep. 2018 Dec 21;8:18045. doi: 10.1038/s41598-018-35936-w (PMC6303410; doi:10.1038/s41598-018-35936-w)
Supplement: Supplementary file 1 — Supplementary Information [file 41598_2018_35936_MOESM1_ESM.pdf]

Supplementary Information

Flavin-based metabolic cycles are integral features of growth and division in single yeast cells

Bridget L. Baumgartner[1,+], Richard O'Laughlin[2,+], Meng Jin[3,+], Lev Tsimring[3], Nan Hao[4], and Jeff Hasty[2,3,4,*]

[1] Booz Allen Hamilton, 8283 Greensboro Drive, Hamilton Building, McLean, VA 22102 USA
[2]University of California San Diego, Department of Bioengineering, La Jolla, CA, 92093, USA
[3]BioCircuits Institute, University of California, San Diego, La Jolla, California, USA
[4]Molecular Biology Section, Division of Biological Science, University of California, San Diego, La Jolla, California, USA

*hasty@bioeng.ucsd.edu
+these authors contributed equally to this work

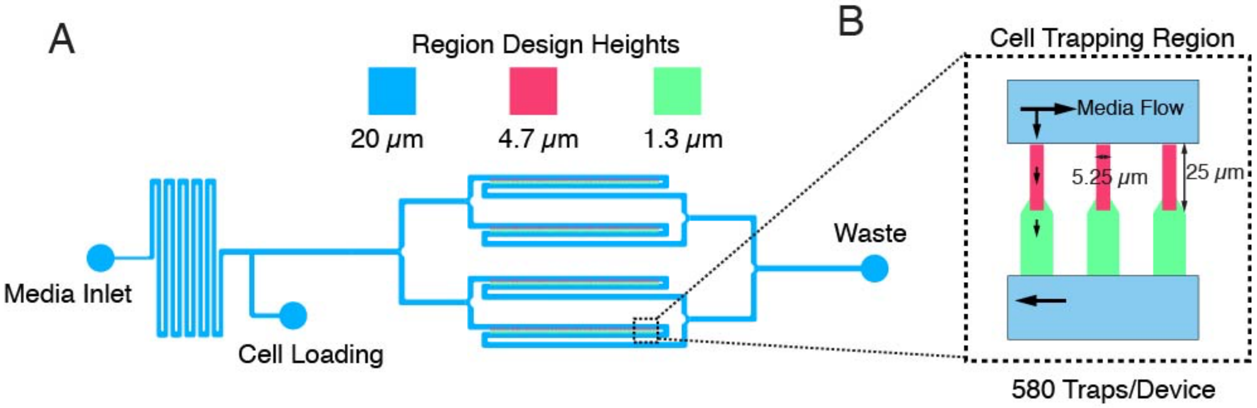

Figure S1. Microfluidic device used in this study. (**A**) Four parallel branches contain cell trapping regions of ~4.7 µm tall (red) and media conduits (~1.3 µm tall), with a total of 580 traps on the entire device. (**B**) This design permits the flow of media from the main channel (~20 µm tall, blue) through the cell trapping region but does not allow cells to pass through the media conduits as they are too low in height. In this way cells are stably maintained at the bottom of the trap (for as long as they bud up toward the entrance of the trap) while constantly being supplied media throughout the course of the experiment. Only one cell is analyzed per trap.

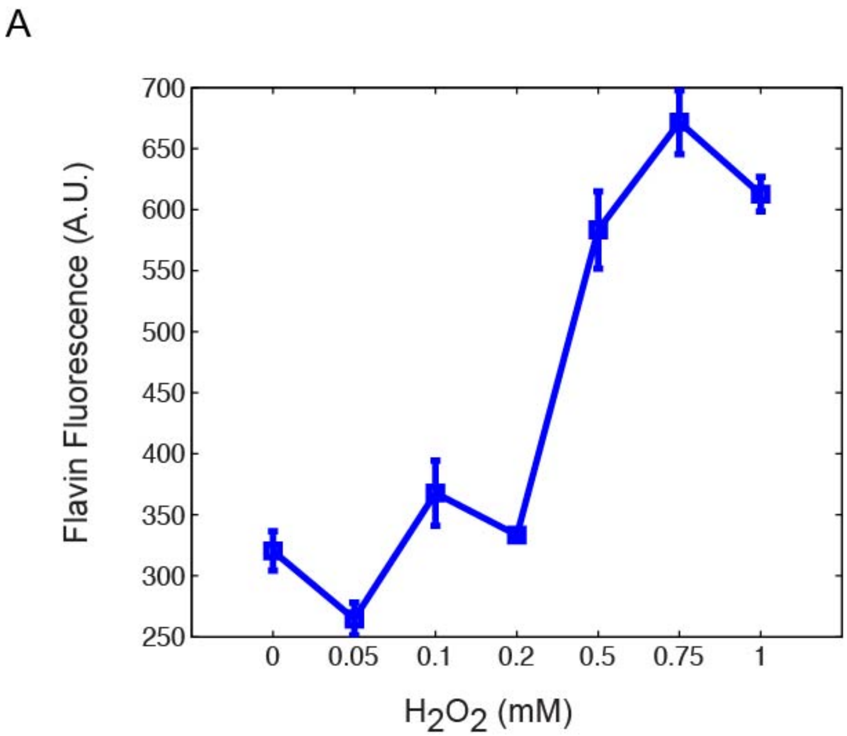

Figure S2. Dynamic range of flavin fluorescence. (**A**) Dose response of flavin fluorescence intensity over a range of H_2O_2 concentrations. Cells were treated with various concentrations of H_2O_2 for 20 minutes after reaching log phase in 1X YNB. Cells were imaged for flavin fluorescence under coverslips. The number of cells collected for each condition is as follows: n = 41 (0 mM), n = 64 (0.05mM), n = 61 (0.1 mM), n = 106 (0.2 mM), n = 30 (0.5 mM), n = 38 (0.75 mM) and n = 101 (1 mM). Error bars are standard error of the mean of measurements from individual cells.

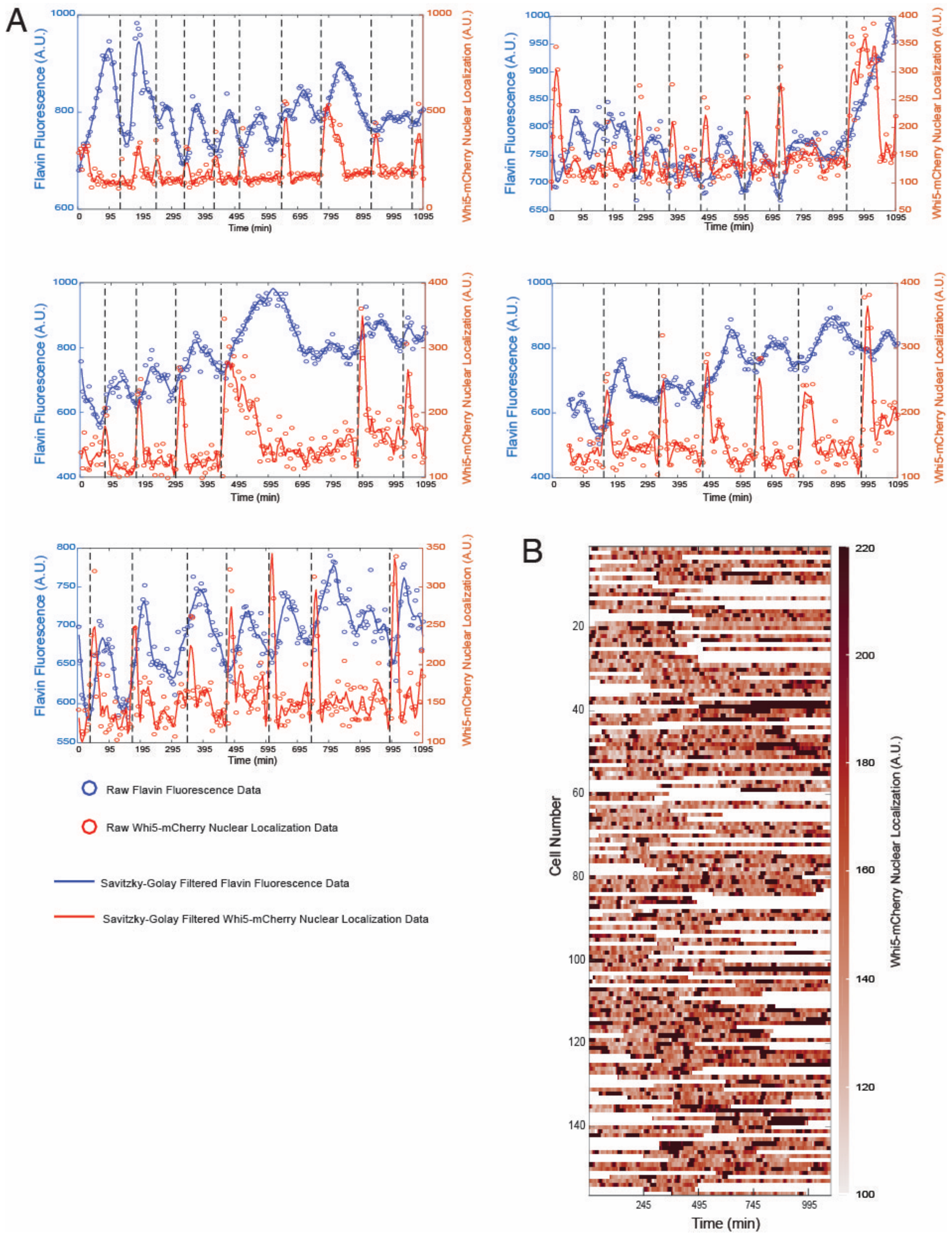

Figure S3. Trajectories of flavin fluorescence and Whi5-mCherry reporter. (**A**) Sample trajectories of flavin fluorescence and the Whi5-mCherry nuclear localization signal. (**B**) Heatmap of Whi5-mCherry nuclear localization in all cells, demonstrating lack of cell division cycle synchrony. The black dotted lines represent separation of the mother and daughter nuclei (from Nhp6a-iRFP reporter).

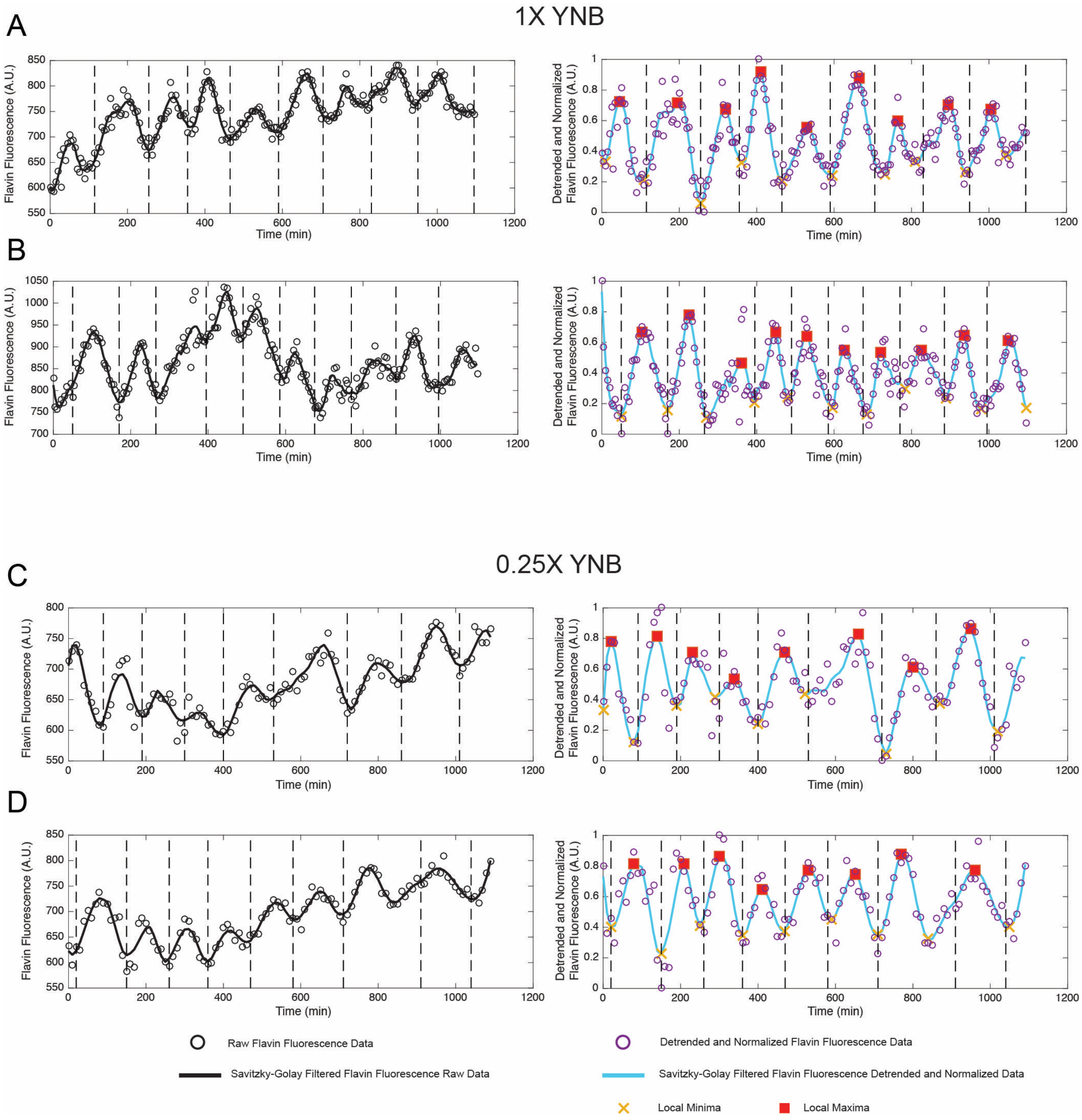

Figure S4. Metabolic cycles in 1X and 0.25X YNB concentrations. (**A**) Sample trajectory for 1X YNB media. (**B**) Additional sample trajectory for 1X YNB media. (**C**) Sample trajectory for 0.25X YNB. (**D**) Additional sample trajectory for 0.25X YNB media. For all detrended and normalized trajectories the red squares represent recorded local YMC peaks, the yellow 'X' marks local metabolic cycle troughs and the black dotted lines represent separation of the mother and daughter nuclei as determined by the Nhp6a-iRFP reporter. In all panels, the raw data and Savitzky-Golay filtered data are shown on the left and the corresponding detrended and normalized trajectory and filtered trajectory are shown on the right.

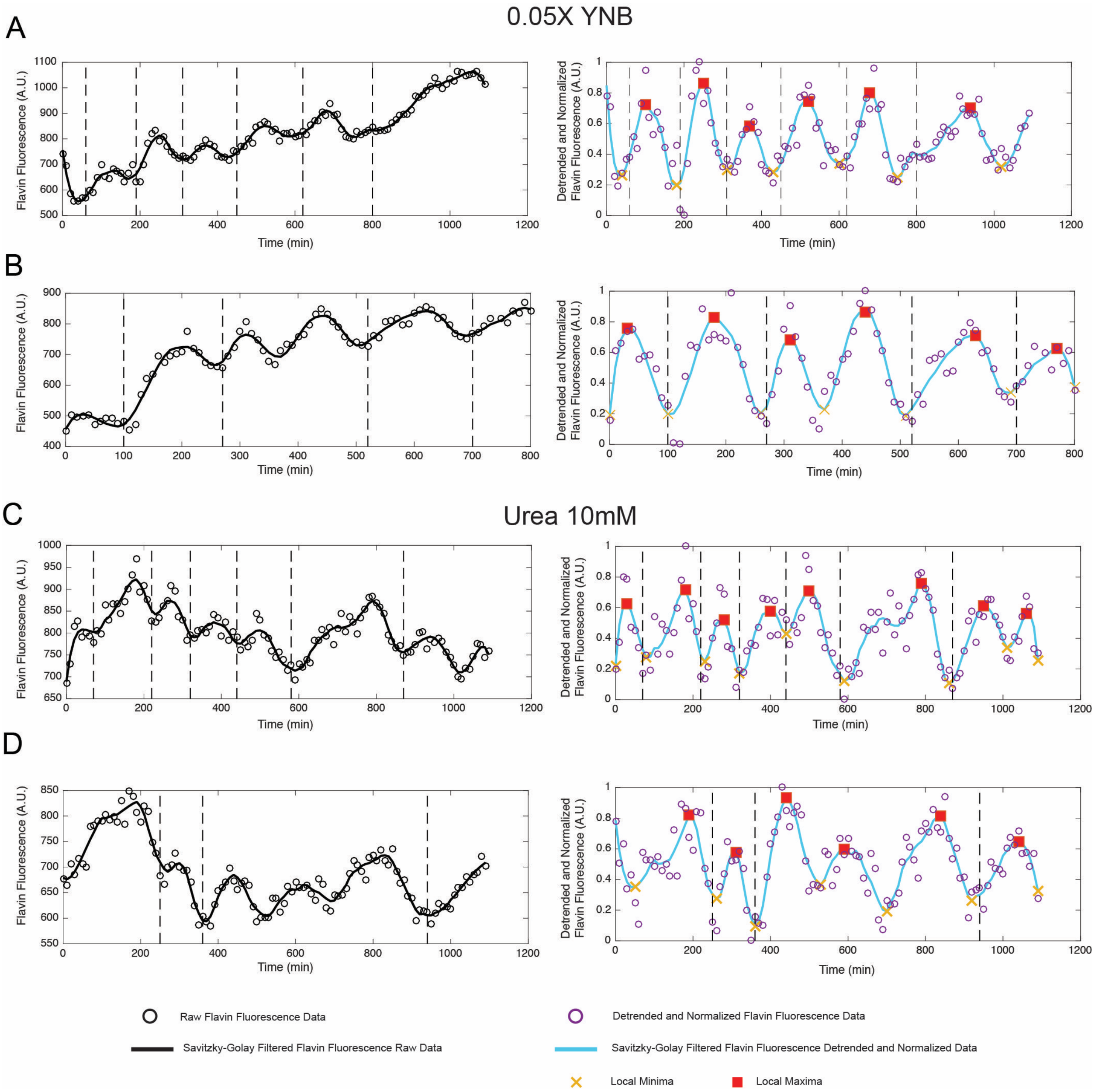

Figure S5. Metabolic cycles in 0.05X YNB and 1X YNB with 10mM urea as the nitrogen source. (**A**) Sample trajectory for 0.05X YNB media. (**B**) Additional sample trajectory for 0.05X YNB media. (**C**) Sample trajectory for 1X YNB with 10mM urea. (**D**) Additional sample trajectory for 1X YNB with 10mM urea. As in Fig. S4, raw data and Savitzky-Golay filtered data are shown on the left and the corresponding detrended and normalized trajectory and filtered trajectory are shown on the right.

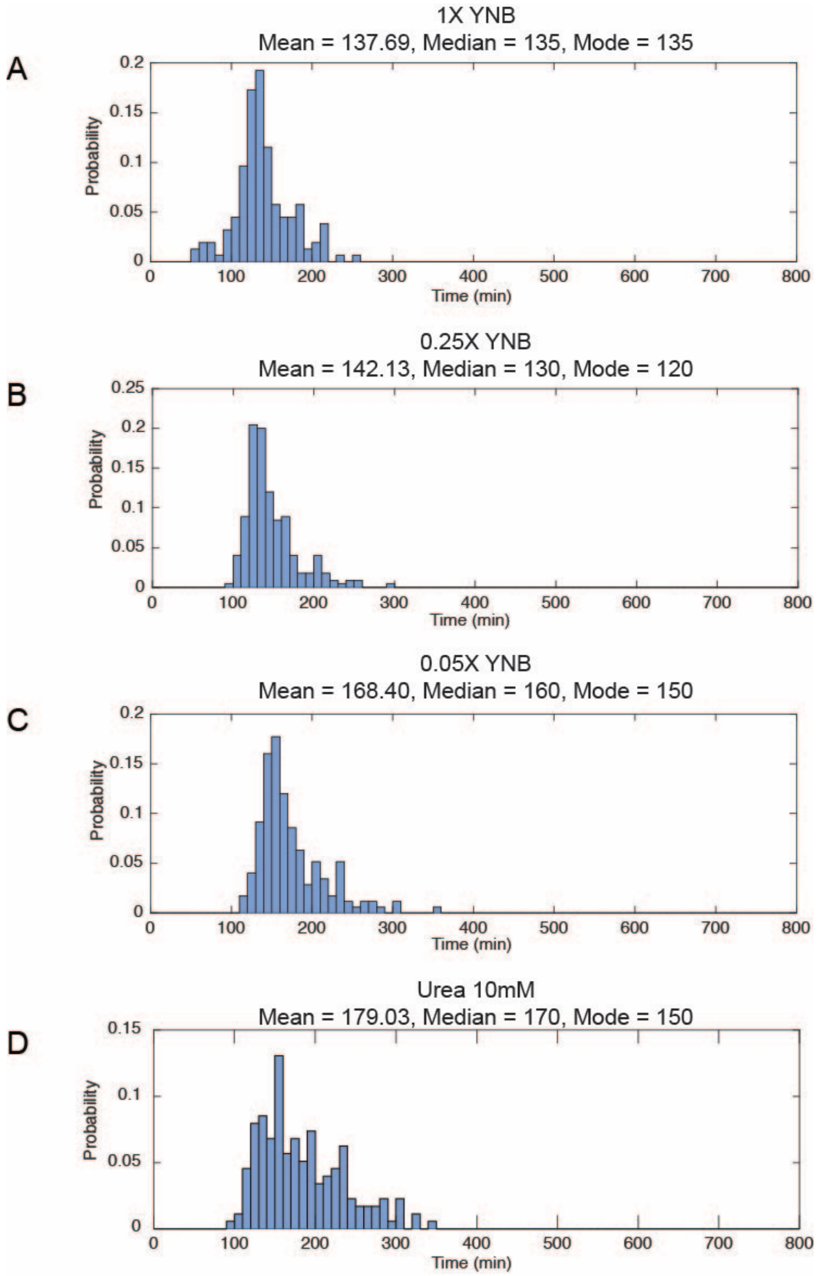

Figure S6. Autocorrelation analysis of metabolic cycles. Summaries of the location of the first autocorrelation peak for each cell for the (**A**) 1X YNB, (**B**) 0.25X YNB and (**C**) 0.05X YNB (**D**) 1X YNB with 10mM urea media conditions.

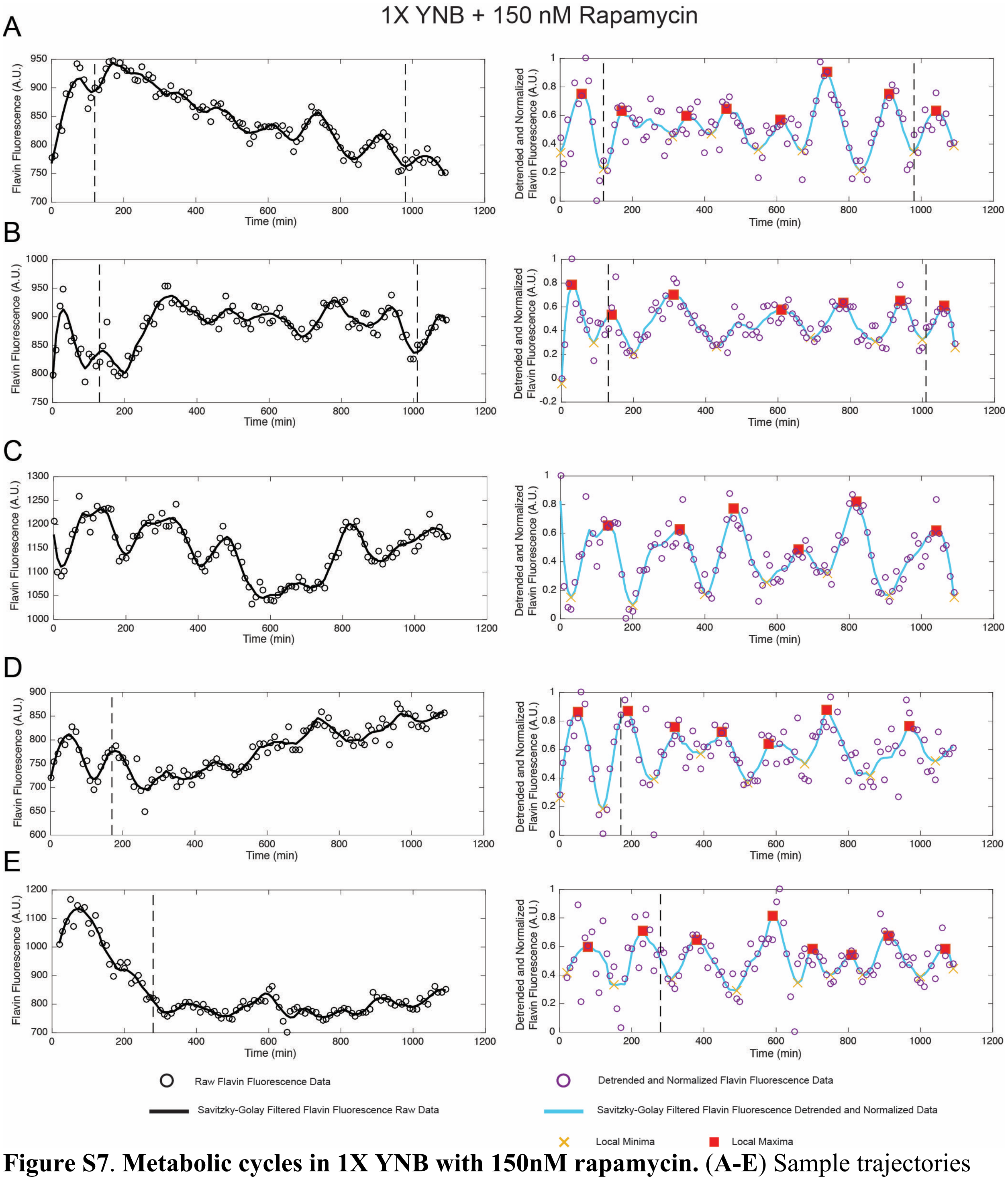

Figure S7. **Metabolic cycles in 1X YNB with 150nM rapamycin.** (**A-E**) Sample trajectories for 1X YNB with 150nM rapamycin. As in Fig. S4 and Fig. S5, raw data and Savitzky-Golay filtered data are shown on the left and the corresponding detrended and normalized trajectory and filtered trajectory are shown on the right.

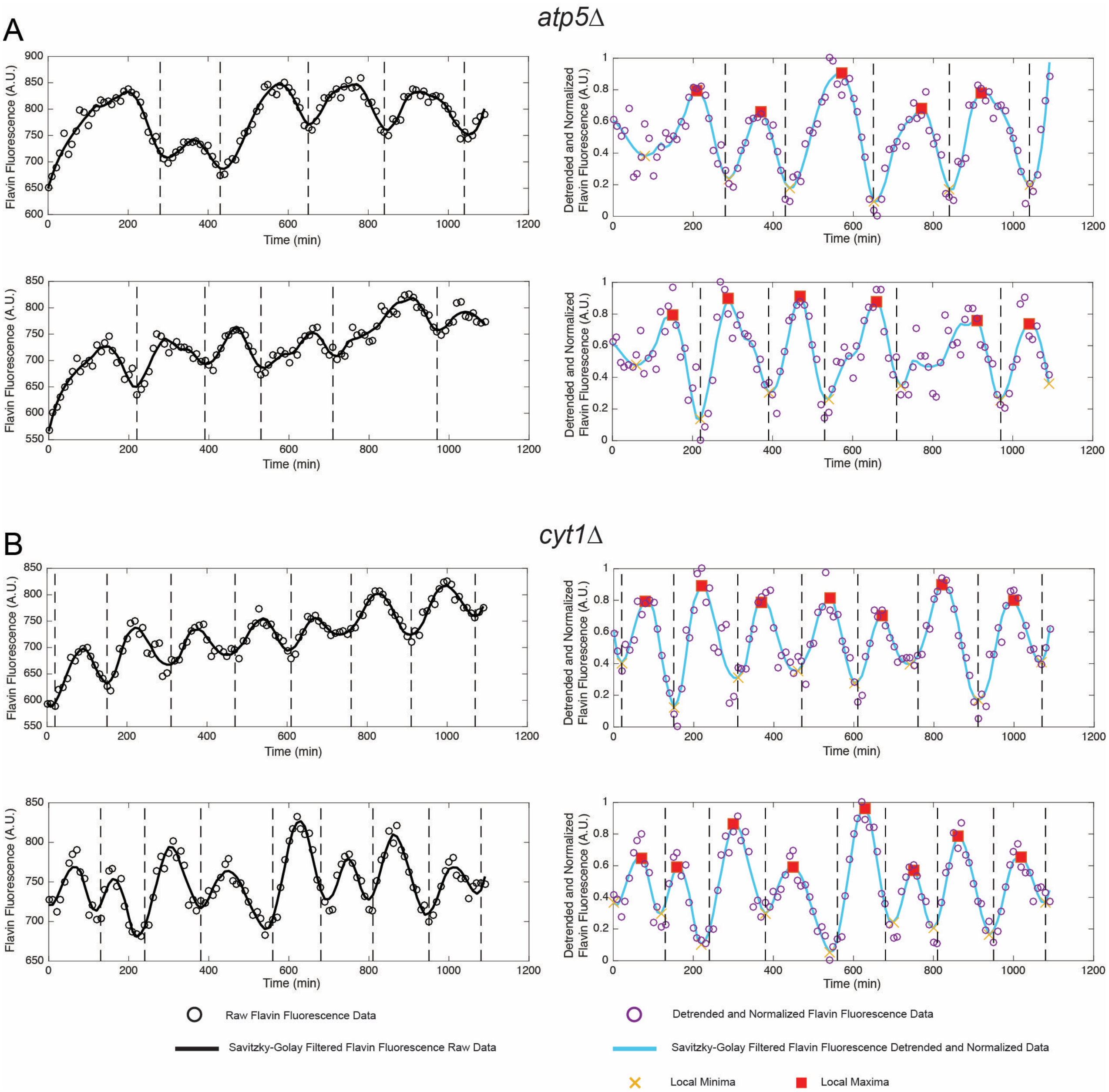

Figure S8. Metabolic cycles in the *atp5*Δ and *cyt1*Δ mutants. (**A**) Sample trajectories for *atp5*Δ. (**B**) Sample trajectories for *cyt1*Δ. Raw data and Savitzky-Golay filtered data are shown on the left and the corresponding detrended and normalized trajectory and filtered trajectory is shown on the right.

Table S1. Parent strain, strains constructed and strains used in experiments. To repair the histidine, leucine and uracil auxotrophies the parent strain CEN.PK2-1c was transformed with standard yeast integration plasmids pRS403 (for repair of histidine auxotrophy), pRS405 (for repair of leucine auxotrophy), or pRS406 (for repair of uracil auxotrophy) where indicated below. To repair the tryptophan auxotrophy we used restriction enzymes to swap a Trp1 selection marker from a pFA6 plasmid into a pKT-mCherry plasmid, replacing the kanMX marker. We then PCR amplified the mCherry-Trp1 fragment and inserted it at the C-terminal end of the *WHI5* gene using standard lithium acetate transformation. Similarly, iRFP-kanMX was PCR amplified and inserted at the C-terminal end of the *NHP6a* locus. Colony PCR was used to verify insertions. To create the respiratory deficient deletion mutants the hphMX marker was PCR amplified from plasmid pAG32 and the natNT2 marker was amplified from plasmid pFA6-natNT2. Colony PCR was used to verify deletions.

Strain Name	Genotype	Additional Details
CEN.PK2-1c	MATa; his3D1; leu2-3_112; ura3-52; trp1-289; MAL2-8c; SUC2	Parent strain. Purchased from EUROSCARF (Accession Number: 30000A)
yBB107	CEN.PK2-1c his3::pRS403, leu2::pRS405, ura3::pRS406	Constructed for this study
yBB108	CEN.PK2-1c leu2::pRS405, ura3::pRS406	Constructed for this study
yBB109	CEN.PK2-1c his3::pRS403, leu2::pRS405	Constructed for this study
yYMC1	CEN.PK2-1c his3::pRS403, leu2::pRS405, ura3::pRS406, Nhp6a-iRFP-kanMX, Whi5-mCherry-Trp1	Constructed for this study
yYMC4	CEN.PK2-1c his3::pRS403, leu2::pRS405, ura3::pRS406, Nhp6a-iRFP-kanMX, Whi5-mCherry-Trp1, *cyt*1::hphMX	Constructed for this study
yYMC5	CEN.PK2-1c his3::pRS403, leu2::pRS405, ura3::pRS406, Nhp6a-iRFP-kanMX, Whi5-mCherry-Trp1, *atp*5::natNT2	Constructed for this study

Table S2. Parameters for metabolic cycle peak detection. In order to quantify properties of the metabolic cycles we wrote custom MATLAB code that allowed peak and minima detection in metabolic cycles using a fixed set of criteria across all experiments. First, the data was detrended by fitting and then subtracting a polynomial of order 7 from the raw data. Qualitatively, the results did not depend on the order of polynomial used for detrending. In general, lower order polynomials failed to remove all baseline drift in the signal, which made applying uniform peak and minima detection difficult across different experiments, while higher order polynomials gave a very even baseline but ran the risk of adding peaks to a signal by being overly sensitive to noise. We found order 7 to be the best balance between these two concerns for our data. After the signal was detrended each signal was normalized according to its maximum and minimum value to between 0 and 1. A Savitsky-Golay filter of order 4 and frame length 17 was then applied to each signal. The MATLAB function 'findpeaks' was used on the filtered signals to detect peaks and mimima according to the specifications listed in the table below.

Order of polynomial used for detrending	7
Savitsky-Golay filter order, frame length	4, 17
Amplitude Threshold	0.09
Minimum distance between peaks	65 min
Minimum peak prominence	0.09
Minimum minima prominence	0.045
Minimum distance between	50 min

Table S3. Metabolic cycle and cell division cycle periods for all experiments. The mean peak-to-peak metabolic cycle periods and CDC periods (calculated from successive nuclear division events as visualized by the Nhp6a-iRFP marker) are displayed for each experiment below. In some media conditions, there were cells that we tracked for only two cell divisions but during one of these divisions, our peak finding code did not detect a peak during one of the divisions. Therefore for such cells a peak-to-peak period could not be calculated, but a CDC period could; hence the one or two cell discrepancy in some cases. For the rapamycin experiment the difference in the number of cells used for metabolic cycle and CDC period calculation is due to the fact that some cells did not divide twice during the experiment, thus a CDC period was not able to be calculated.

**Experiment**	**Metabolic Cycle Period Mean (min)**	**Metabolic Cycle Period Standard Deviation (min)**	**Cell Division Cycle Mean (min)**	**Cell Division Cycle Standard Deviation (min)**	**Total Number of Cells for Calculation (Metabolic Cycle, CDC)**
**1X YNB**	135.91	40.56	142.53	45.95	155, 156
**0.25X YNB**	136.43	36.11	138.30	40.93	224, 225
**0.05X YNB**	162.80	47.52	178.83	63.73	174, 175
**1X YNB with 10mM urea**	161.48	54.52	181.19	90.49	174, 176
**1X YNB+150nM rapamycin**	191.73	75.41	416.94	238.51	180, 85
***atp5*Δ, 1X YNB**	186.58	54.37	218.44	65.40	54, 54
***cyt1*Δ, 1X YNB**	162.81	42.04	168.70	53.20	51, 52
